# The roles of circRFWD2 and circINO80 during NELL‐1‐induced osteogenesis

**DOI:** 10.1111/jcmm.14726

**Published:** 2019-10-21

**Authors:** Xinqi Huang, Xiao Cen, Bo Zhang, Yuwei Liao, Zhenxing Zhao, Guanyin Zhu, Zhihe Zhao, Jun Liu

**Affiliations:** ^1^ State Key Laboratory of Oral Diseases & National Clinical Research Center for Oral Diseases West China Hospital of Stomatology Sichuan University Chengdu China; ^2^ Department of Orthodontics West China Hospital of Stomatology Sichuan University Chengdu China; ^3^ Department of Temporomandibular Joint West China Hospital of Stomatology Sichuan University Chengdu China

**Keywords:** circINO80, circRFWD2, hsa‐miR‐6817‐5p, NELL‐1, osteogenic differentiation

## Abstract

Bone defects caused heavy social and economic burdens worldwide. Nel‐like molecule, type 1 (NELL‐1) could enhance the osteogenesis and the repairment of bone defects, while the specific mechanism remains to be elucidated. Circular RNAs (circRNAs) have been found to play critical roles in the tissue development and serve as biomarkers for various diseases. However, it remains unclear that the expression patterns of circRNAs and the roles of them played in recombinant NELL‐1‐induced osteogenesis of human adipose‐derived stem cells (hASCs). In this study, we performed RNA‐sequencing to investigate the expression profiles of circRNAs in recombinant NELL‐1‐induced osteogenic differentiation and identified two key circRNAs, namely circRFWD2 and circINO80. These two circRNAs were confirmed to be up‐regulated during recombinant NELL‐1‐induced osteogenesis, and knockdown of them affected the positive effect of NELL‐1 on osteogenesis. CircRFWD2 and circINO80 could interact with hsa‐miR‐6817‐5p, which could inhibit the osteogenesis. Silencing hsa‐miR‐6817‐5p could partially reverse the negative effect of si‐circRFWD2 and si‐circINO80 on the osteogenesis. Therefore, circRFWD2 and circINO80 could regulate the expression of hsa‐miR‐6817‐5p and influence the recombinant NELL‐1‐induced osteogenic differentiation of hASCs. It opens a new window to better understanding the effects of NELL‐1 on the osteogenic differentiation of hASCs and provides potential molecular targets and novel methods for bone regeneration efficiently and safely.

## 1 INTRODUCTION

Bone defects, which caused by congenital diseases, trauma and surgeries, have led to enormous burdens and affect the people's quality of life seriously, but the treatment of critical bone defects remains a difficult biomedical problem.^1^ The autogenous bone is considered as the gold standard and performed preferentially.^2^, ^3^ This procedure, however, could not satisfy the increasing need of repairing the large bone defects, because of the distinct donor‐site morbidity and limited volume.^4^ As the alternative approach, xenografts can cause immunological rejection and pathogen transmission.^5^ Bone tissue engineering with potent growth factors has emerged as a promising method in bone regeneration.^6^, ^7^ Human adipose‐derived stem cells (hASCs), acting as the seed cells, have received widespread attention as a result of their minimally invasive acquisition and the ability of multiple differentiation.^8^, ^9^ How to induce the osteogenesis of hASCs effectively and safely, however, remains a research focus.

Nel‐like molecule, type 1 (NELL‐1), as a secreted osteogenic growth factor, was detected initially in patients diagnosed with unilateral coronal synostosis.^10^ Nell‐1 deficiency mice revealed major skeletal anomalies, including decreased calvaria mineralization, depressed vertebral disc and even perinatal death.^11^ In turn, the overexpression of NELL‐1 could promote the bone formation in vitro and in vivo models.^12^, ^13^, ^14^ It is reported that NELL‐1 was not only specific to the osteochondral lineage,^15^, ^16^ but also served as a critical functional mediator in the downstream of Runx2 which was essential for osteoblast differentiation.^17^ These factors may contribute to fewer complications of NELL‐1‐induced osteogenesis, especially in the heterotopic bone formation, while the specific mechanism remains to be elucidated.

Circular RNAs (circRNAs) are generated by splicing during pre‐mRNAs maturity. Unlike linear RNAs, they hold a covalently closed loop, keeping them stable and resistant to RNase R digestion.^18^, ^19^ High throughput sequencing has demonstrated that circRNAs were widely and abundantly distributed in the eukaryotic transcriptome with tissue‐specific expression.^20^ Functionally, circRNAs could regulate the protein function via cap‐independent mechanism, affect the splicing of parental genes and serve as the microRNAs (miRNAs) sponges to cross‐talk with miRNAs.^21^, ^22^, ^23^ Emerging evidence has proven that circRNAs played important roles in the development of normal tissues and various diseases, such as cardiovascular diseases, age‐related diseases, autoimmune diseases and tumours.^24^, ^25^, ^26^, ^27^, ^28^, ^29^, ^30^ Their functions as potential biomarkers were gradually discovered in cancers and neurodegenerative disorders.^31^, ^32^ However, it remains unclear that the comprehensive expression patterns of circRNAs and the roles of them played in the NELL‐1‐induced osteogenesis of hASCs.

In present study, we performed RNA‐sequencing and compared the expression profiles of circRNAs between recombinant NELL‐1‐induced osteogenic differentiation group (NG) and general osteogenic differentiation group (OG). The potential genomic function was investigated by Gene ontology (GO) and Kyoto Encyclopedia of Genes and Genomes (KEGG) pathway analyses. Two key circRNAs, namely circRFWD2 and circINO80, were identified to be up‐regulated during recombinant NELL‐1‐induced osteogenesis. Our results indicated that circRFWD2 and circINO80 could regulate the expression of hsa‐miR‐6817‐5p and influence the recombinant NELL‐1‐induced osteogenesis of hASCs.

## 2 MATERIALS AND METHODS

### 2.1 Cell culture and recombinant NELL‐1‐induced osteogenic differentiation of hASCs

The hASCs were purchased from Cyagen company. All the hASCs were separated into two groups: recombinant NELL‐1‐induced osteogenesis group (NG) and general osteogenesis group (OG).

The OriCell™ general medium, which was utilized to culture hASCs, contained 10% mesenchymal stem cell‐qualified foetal bovine serum (FBS), 1% glutamine and 1% penicillin‐streptomycin. The osteogenic induction was initiated once the confluence of hASCs reached 80%. The general osteogenic inductive medium included basic medium, 10 nM dexamethasone, 10 mM β‐glycerophosphate and 50 μg/mL vitamin C (Sigma‐Aldrich), which was applied to culture the hASCs in OG. The hASCs in NG was culture in the general osteogenic inductive medium supplemented with 300 ng/mL of recombinant human NELL‐1 protein (R&D Systems).^33^ The cells were maintained in an incubator with 5% CO_2_ at 37℃.

### 2.2 Alizarin red s (ARS) staining

Alizarin red s staining was performed to detect the mineral deposition. Based on the protocol, the samples were fixed in 4% paraformaldehyde solution at 4°C for 20 minutes. After washed three times with distilled water, the samples were stained with 0.1% ARS for 20 minutes at 25℃.

### 2.3 Alkaline phosphatase (ALP) staining and ALP activity

Alkaline phosphatase staining was implemented with the Leukocyte Alkaline Phosphatase Kit 86C (Sigma) on the basis of the manufacturer's instruction. In brief, samples were washed with PBS and fixed in citrate solution for 30 seconds. Then, they were stained with the solution of FRV alkaline, naphthol AS‐BI alkaline and sodium nitrite for 15 minutes, protected from light during this process.

The Alkaline Phosphatase Assay Kit (Beyotime) was used for the analysis of ALP activity. The samples were cracked with RIPA lysis buffer, and supernatant was transferred to a 96‐well plate. The 96‐well plate was incubated at 37℃ for 30 minutes after p‐nitrophenol, and the reaction substrates were added. Finally, the ALP activity was calculated using p‐nitrophenyl substrate at 405 nm.

### 2.4 CircRNAs sequencing

The total RNAs were isolated from NG and OG with the Trizol reagent (Invitrogen). The RNA purity and concentration of samples were determined by NanoDrop ND‐1000 (NanoDrop Thermo). The RNA integrity of samples was detected by denaturing agarose gel electrophoresis. In general, 3 units/μg of RNase R (Epicentre, Inc) were applied to treat 5μg RNAs for 15 minutes at 37°C to remove linear RNA. The rRNAs of the Rnase R–treated RNAs were depleted using Ribo‐Zero Magnetic Gold Kit (Epicentre, Inc).

### 2.5 CircRNAs profiling analysis

CircRNA sequencing and RNA library construction were performed by CloudSeq Biotech Inc. The RNA libraries were constructed with a TruSeq Stranded Total RNA Library Prep Kit (Illumina). The quality and quantity of RNAs in the libraries were determined by the BioAnalyzer 2100 system (Agilent Technologies). RNA‐Seq sequencing was conducted on the Illumina HiSeq platform. The Illumina HiSeq 4000 sequencer was used to acquire paired‐end reads.

Edger software was utilized to identify the differentially expressed circRNAs among NG and OG.^34^ The hierarchical clustering analysis was performed based on the significant differences. Any circRNAs, presenting fold changes >2.0 with *P* values < .05, were considered as significant differential expression.

The GO and KEGG analyses were used to predict the functions of differentially expressed circRNA‐associated genes. GO analysis measured biological processes, cellular components and molecular functions. KEGG pathway analysis was used to identify pathways related to the target mRNAs of circRNAs. To investigate the potential functions of the differentially expressed circRNAs, the prediction software was utilized to show the interactions of these circRNAs with the targeted miRNAs. The prediction of miRNA‐binding sites of the targeted mRNAs was performed on the basis of TargetScanHuman 7.2 and miRanda.

### 2.6 Cell transfection

The mimic and the inhibitor of hsa‐miR‐6817‐5p, miRNA control, circRFWD2 siRNA, circINO80 siRNA and control vector were synthesized by GenePharma Co. and shown in Table S1. Cells were transfected by Lipofectamine 3000 Reagent (Invitrogen), when the cell density reached 80% confluency.

### 2.7 Quantitative real‐time PCR

For the selected circRNAs, total RNAs (3 μg) were employed for first strand cDNA synthesis with dNTP Mix (HyTestLtd), RNase inhibitor (Enzymatics) and SuperScript III Reverse Transcriptase (Thermo Fisher Scientific). The qRT‐PCR was performed on an Applied Biosystems 7500 Fast Real‐Time PCR System (Applied Biosystems) using SYBR Green master mix (Cloudseq). The primers of circRNAs and genes were synthesized by Sangon and shown in Table S2.

The cDNA synthesis and quantitative detection of miRNAs were performed with the miRNA qRT‐PCR Detection Kit (GeneCopoeia). The primer of hsa‐miR‐6817‐5p was designed by GeneCopoeia. U6 was used for normalization. The relative expression was calculated by the formula 2^−ΔΔCt^.

### 2.8 Western blot

The protein levels of RUNX2 and bone sialoprotein (BSP) were determined by Western blot. Radioimmunoprecipitation assay (RIPA) lysis buffer was used to extract total cell protein. Protein concentration was determined by the BCA Protein Assay Kit (Thermo). Equal microlitres of protein samples were loaded onto sodium dodecyl sulfate‐polyacrylamide gel electrophoresis (SDS‐PAGE), and then, they were transferred onto PVDF membranes (Millipore). The PVDF membranes were incubated with monoclonal antibodies against anti‐RUNX2 (1:1000, CST), anti‐BSP (1:1000, Abcam) and GAPDH (1:1000, Abcam) overnight at 4°C. After washed with TBST, the membranes were incubated with corresponding secondary antibodies (1:5000, Abcam) for 2 hours. The band intensity was determined by ImageJ software. All the target bands were normalized to GAPDH band.

### 2.9 Statistics

Quantitative data were expressed as means ± standard deviation (SD), and all the experiments were performed three times at least. The statistical analysis was performed with SPSS 17.0 software. The differences between two groups were analysed by unpaired t test, while one‐way analysis of variance (ANOVA) were utilized to identify the differences between more than two groups. *P*‐value < .05 was considered as statistical significance.

## 3 RESULTS

### 3.1 Recombinant NELL‐1‐induced osteogenic differentiation of hASCs

**Figure 1:**
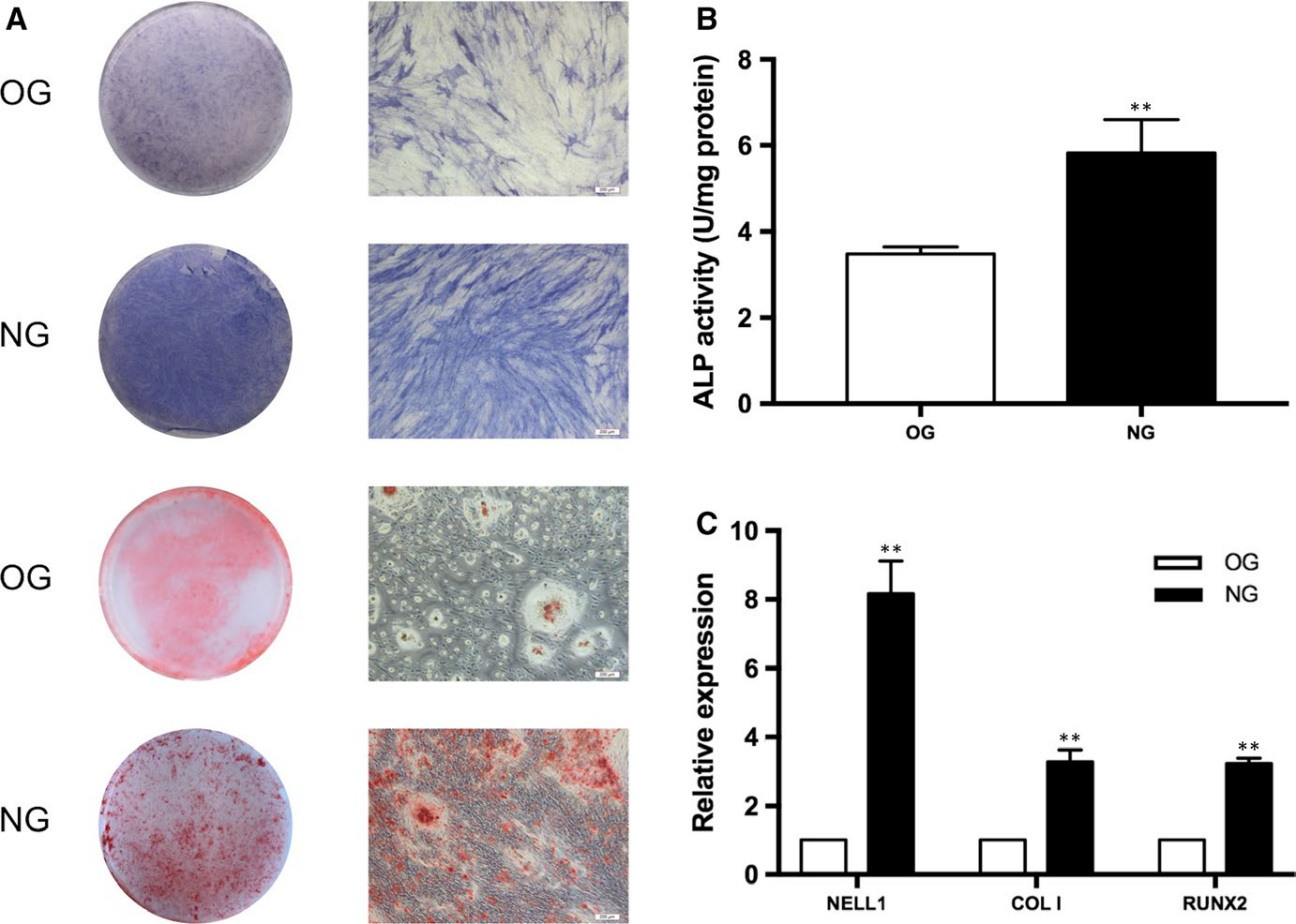
Recombinant NELL‐1‐induced osteogenic differentiation of hASCs. A, ALP staining and ARS staining after 7 d of induction of hASCs. B, Quantification of ALP activity after 7 d of induction of hASCs. C, Real‐time qPCR was used to detect the expressions of NELL‐1, COL I, and RUNX2. **P < .01 (scale bar = 200 μm)

After induced for 7 days, the intensity of ALP staining was much more positive and the calcified nodules of ARS staining were apparently spotted in NG (Figure 1A). The quantification of ALP activity were larger in NG than that in OG (*P* < .01) (Figure 1B).

The expression of NELL‐1 was significantly increased in NG, compared with OG (*P* < .01). Meanwhile, the expression levels of osteogenic markers, namely COL I and RUNX2, were up‐regulated significantly in NG (*P* < .01, *P* < .01, respectively) (Figure 1C). These results above suggested that NELL‐1 could enhance osteogenesis of hASCs.

### 3.2 Expression patterns of circRNAs in recombinant NELL‐1‐induced hASCs

Totally, 13 203 circRNAs were identified in hASCs after recombinant NELL‐1‐induced osteogenic differentiation. Among these, 9438 circRNAs have been recorded in the circBase and/or reported by other studies, while 3765 circRNA were novel in this study.

**Figure 2:**
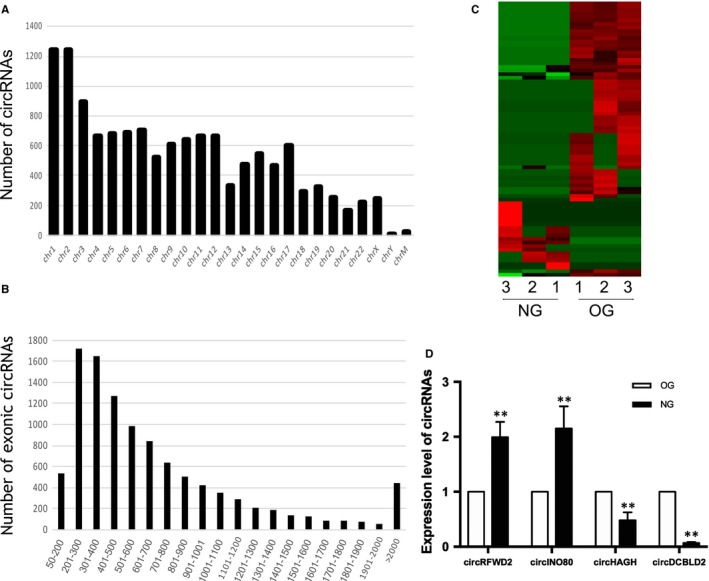
The profile of circRNAs in recombinant NELL‐1‐induced osteogenic differentiation of hASCs. A, Distribution of circRNAs in chromosomes, ‘ChrM’ represents mitochondrial genome. B, The length of 10 624 exonic circRNAs, the majority of them ranged from 201 to 300 nt (16.21%) in size. C, Heat map was performed to assess the differentially expressed circRNAs between OG and NG. D, Real‐time qPCR was utilized to validate the differently expressed circRNAs. **P < .01

The 13 203 circRNAs were distributed across all chromosomes. Chromosomes 1‐22 compromised over 150 circRNAs and X contained 241 circRNAs, while there were less than 20 circRNAs in Y and mitochondrial chromosomes (Figure 2A). The size of 10 624 exonic circRNAs ranged from 53 nt to more than 2000 nt and the majority of them (16.21%) were 201‐300 nt long, while the average length was 773.69 nt (Figure 2B).

The overview of differentially expressed circRNAs between NG and OG was displayed by heatmap after fold changes filtering (Figure 2C). In total, 77 circRNAs were identified to express differentially, among which 19 circRNAs were up‐regulated and 58 circRNAs were down‐regulated significantly. Four circRNAs, namely circRFWD2, circINO80, circHAGH and circDCBLD2, were selected based on their raw intensities, fold changes and *P* values. CircRFWD2 and circINO80 were up‐regulated, while circHAGH and circDCBLD2 were down‐regulated in NG. The results of qRT‐PCR were consistent with RNA‐sequencing (Figure 2D).

### 3.3 GO and KEGG pathways analyses of the host genes of circRNAs

Gene ontology analysis was performed to analyse the host genes of differentially expressed circRNAs. It contained three aspects, that is biological processes, cellular components and molecular function. The top 60 enrichment GO analysis was shown in Figure S1. The most enriched biological processes terms were associated with the regulation of cell cycle process (GO:0010564), the organelle organization (GO:0006996) and the mitotic nuclear division (GO:0007067). The most enriched cellular components terms were the intracellular part (GO:0044424), the nucleoplasm (GO:0005654) and the intracellular (GO:0005622). For molecular function aspect, the most enriched terms were related to the protein binding (GO:0005515), the guanyl‐nucleotide exchange factor activity (GO:0005085) and the small GTPase binding (GO:0031267).

As for the KEGG pathway analysis, the top 39 pathways were listed according to the enrichment scores (Figure S2). The most enriched pathways included Glutamatergic synapse (hsa04724), Thyroid hormones (THs) signalling pathway (hsa04919) and p53 signalling pathway (hsa04115).

### 3.4 The effects of circRFWD2 and circINO80 on osteogenesis

**Figure 3:**
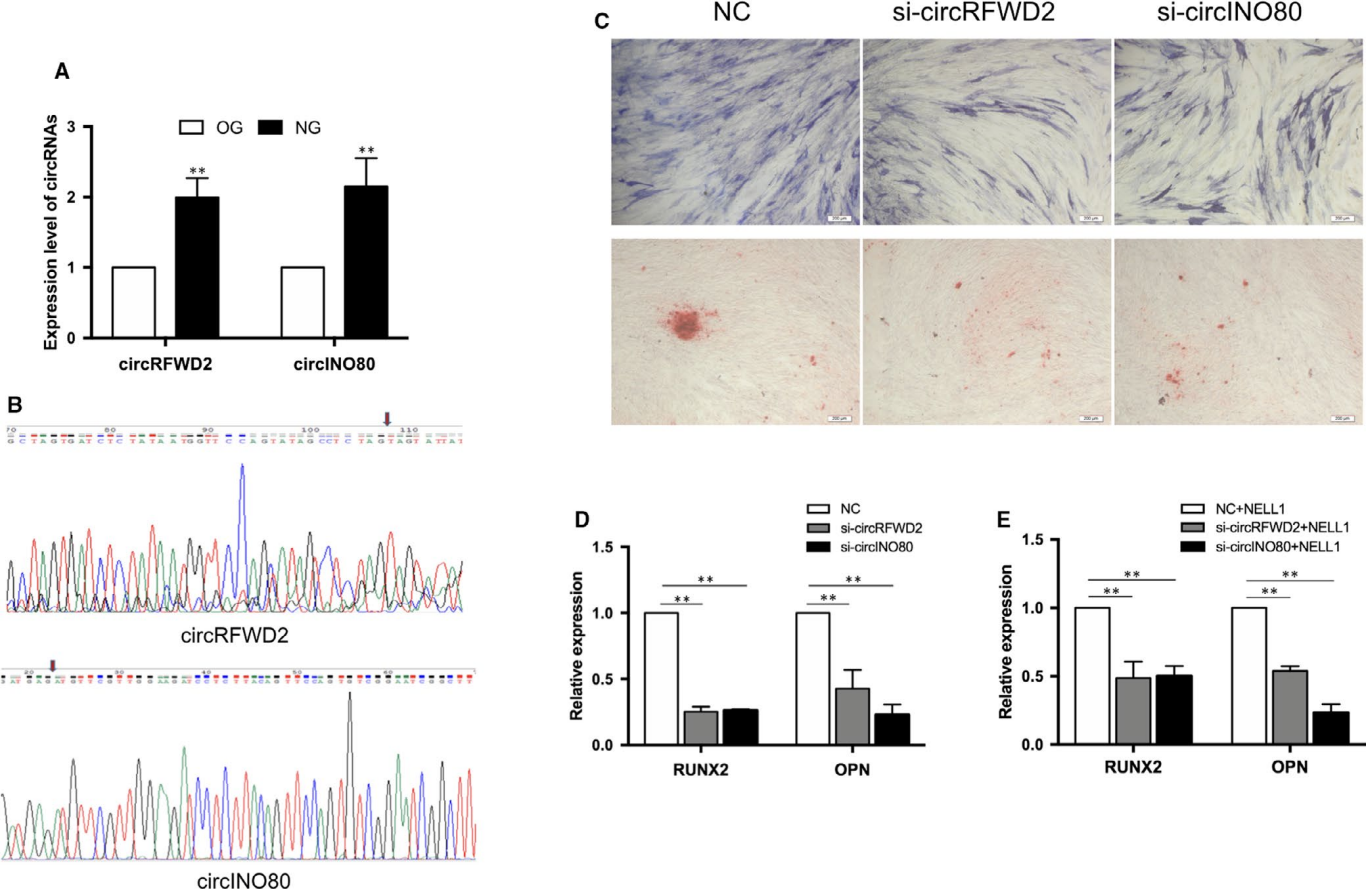
The effects of circRFWD2 and circINO80 on osteogenesis. A, Real‐time qPCR was used to determine the expression levels of circRFWD2 and circINO80. B, Sanger Sequencing results of circRFWD and circINO80. C, ALP and ARS staining after 7 d of induction in si‐circRFWD2 and si‐circINO80 groups. D, Real‐time qPCR was used to determine the expression levels of RUNX2 and OPN in si‐circRFWD2 and si‐circINO80 groups. E, Real‐time qPCR was used to determine the expression levels of RUNX2 and OPN in si‐circRFWD2 and si‐circINO80 groups with NELL‐1 induction. **P < .01 (scale bar = 200 μm)

After induced for 7 days, the expressions of circRFWD2 and circINO80 were up‐regulated in NG, when were compared with those in OG (Figure 3A). Sanger sequencing confirmed the specific splicing sites of circRFWD2 and circINO80 (Figure 3B).

Transfection was conducted to knock down the expression of circRFWD2 and circINO80. After osteogenic induction for 7 days, the intensity of ALP and ARS staining decreased in both of circRFWD2 and circINO80 knockdown groups (Figure 3C). The outcomes of qRT‐PCR indicated that the expression levels of RUNX2 and OPN were decreased in both of circRFWD2 and circINO80 knockdown groups, when compared to group NC (Figure 3D).

To investigate the function of circRFWD2 and circINO80 on recombinant NELL‐1‐induced osteogenesis, the expressions of osteogenic markers were detected in circRFWD2 and circINO80 knockdown groups, respectively, after recombinant NELL‐1‐induced osteogenic differentiation. It showed that the expression levels of RUNX2 and OPN were reduced significantly in both of circRFWD2 and circINO80 knockdown groups, when compared with group NC (Figure 3E).

### 3.5 The co‐targeted miRNAs of circRFWD2 and circINO80

**Figure 4:**
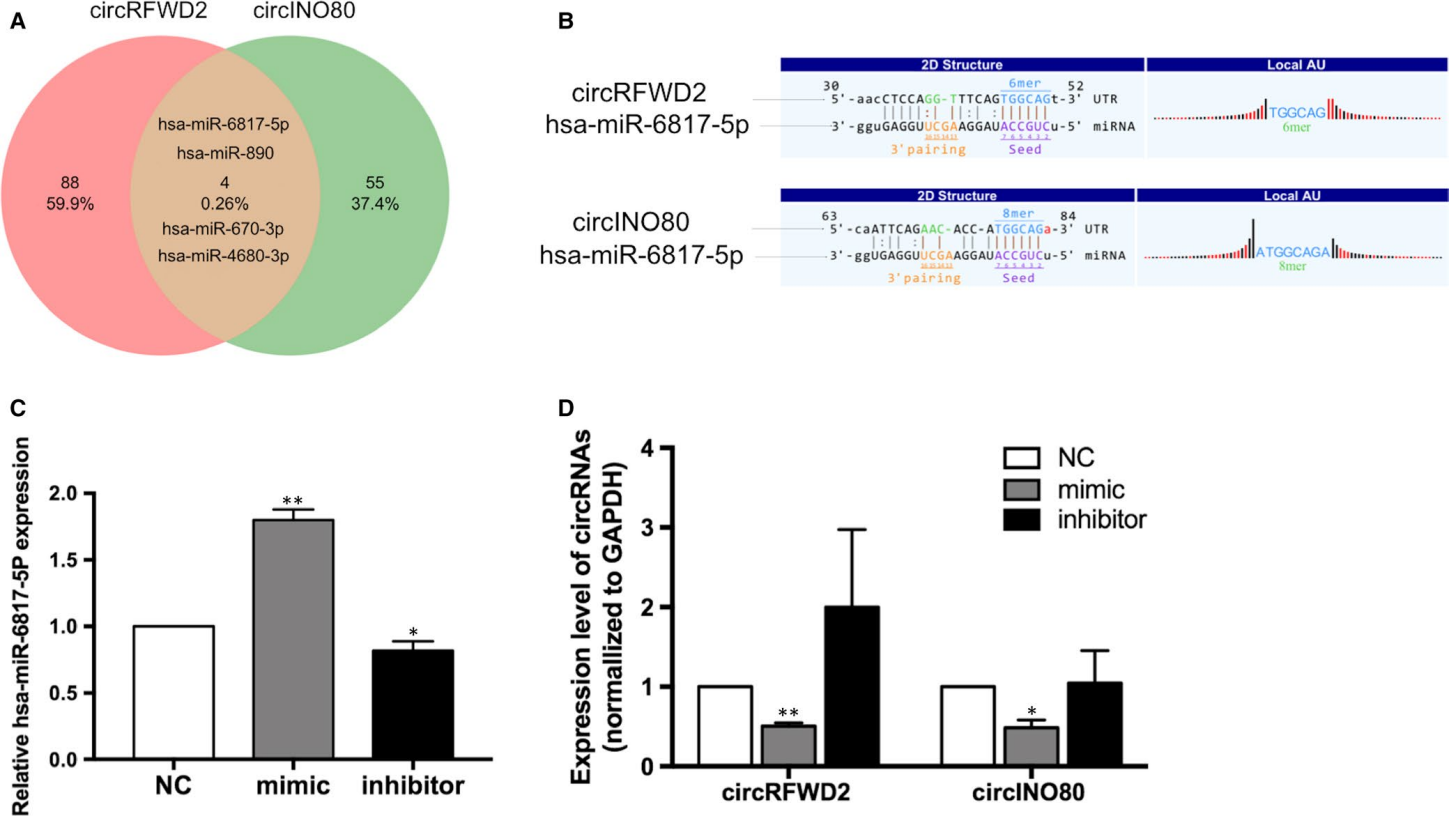
The co‐targeted miRNAs of circRFWD2 and circINO80. A, Co‐targeted miRNAs of circRFWD2 and circINO80. B, The binding sites of hsa‐miR‐6817‐5p in circRFWD2 and circINO80. C, Real‐time qPCR detected the expression level of hsa‐miR‐6817‐5p in mimic, inhibitor, and NC group. D, Real‐time qPCR detected the expressions of circRFWD2 and circINO80 in mimic, inhibitor and NC groups. *P < .05, **P < .01

According to miRanda and TargetScanHuman 7.2 database, we obtained four co‐targeted miRNAs of circRFWD2 and circINO80, including hsa‐miR‐890, hsa‐miR‐670‐3p, hsa‐miR‐4680‐3p and hsa‐miR‐6817‐5p (Figure 4A). It was predicted that circRFWD2 and circINO80 possessed one miRNA‐binding site for hsa‐miR‐6817‐5p, respectively (Figure 4B).

After transfected with hsa‐miR‐6817‐5p mimic and hsa‐miR‐6817‐5p inhibitor for 7 days, the expression level of hsa‐miR‐6817‐5p was increased in mimic group (*P* < .01) and decreased in inhibitor group significantly (*P* < .05) (Figure 4C). Meanwhile, the expression levels of circRFWD2 and circINO80 were down‐regulated significantly in hsa‐miR‐6817‐5p mimic group (*P* < .01, *P* < .05, respectively), and those circRNAs were up‐regulated in hsa‐miR‐6817‐5p inhibitor group without significance (Figure 4D).

### 3.6 The effects of hsa‐miR‐6817‐5p on osteogenesis

**Figure 5:**
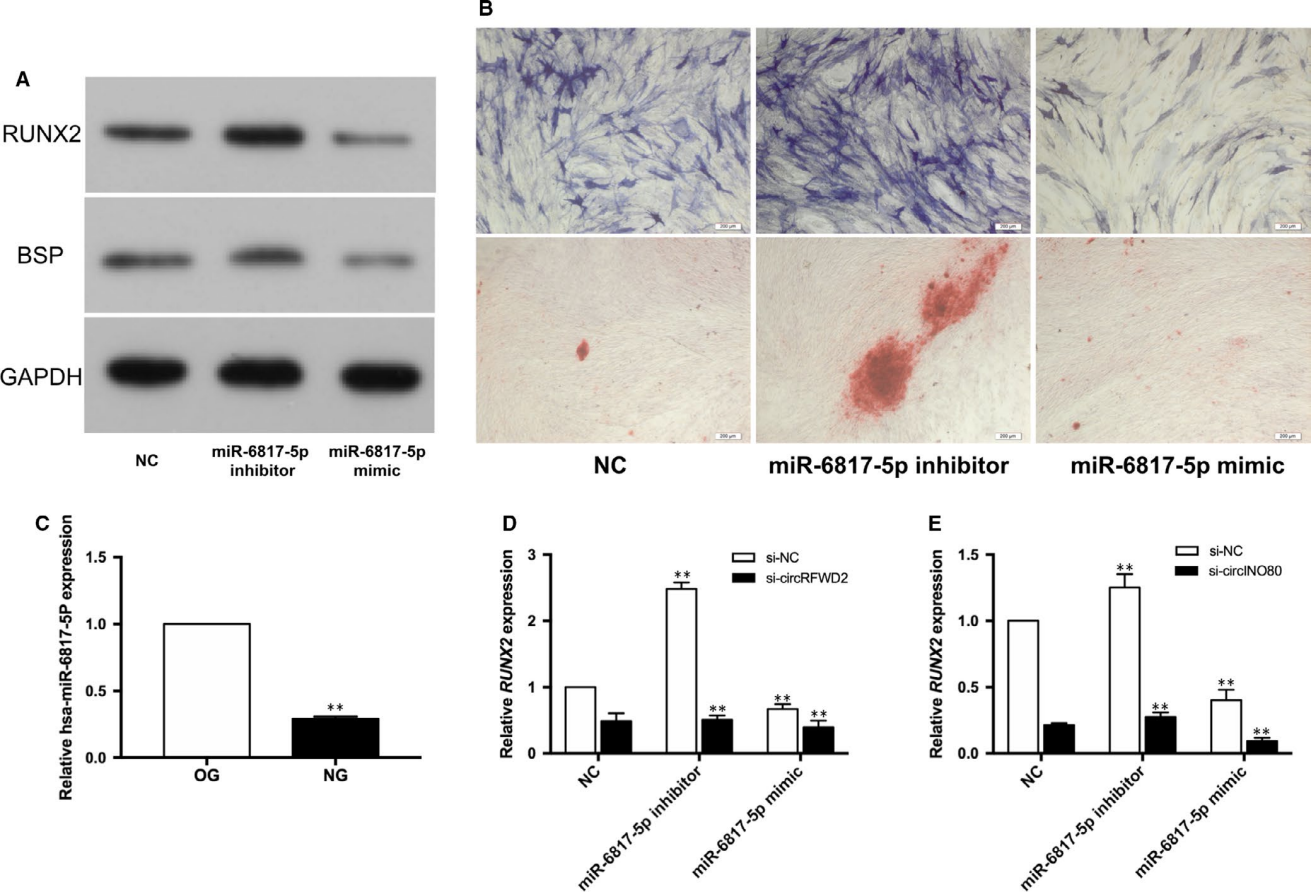
The effects of hsa‐miR‐6817‐5p on the osteogenesis of hASCs. A, Western blot detected the protein levels of RUNX2 and BSP in hsa‐miR‐6817‐5p mimic, inhibitor and NC groups. B, The ALP and ARS staining of hASCs in hsa‐miR‐6817‐5p mimic, inhibitor and NC groups. C, Real‐time qPCR detected the expression of hsa‐miR‐6817‐5p in OG and NG. D, The expression levels of RUNX2 of hASCs after co‐transfection with si‐NC or si‐circRFWD2 plus hsa‐miR‐6817‐5p mimic, inhibitor or NC groups. E, The expression levels of RUNX2 of hASCs after co‐transfection with si‐NC or si‐ circINO80 plus hsa‐miR‐6817‐5p mimic, inhibitor or NC groups. **P < .01. (scale bar = 200 μm)

After transfected with hsa‐miR‐6817‐5p mimic, the protein levels of BSP and RUNX2 were down‐regulated markedly (Figure 5A), and the intensity of ALP staining and ARS staining were obviously weaker in the mimic group (Figure 5B). When inhibiting hsa‐miR‐6817‐5p, we found the expression of BSP and RUNX2 were up‐regulated (Figure 5A). ALP staining and ARS staining were more positive in the inhibitor group (Figure 5B).

After induced for 7 days, the expression level of hsa‐miR‐6817‐5p was lower significantly in NG than that in OG (*P* < .01) (Figure 5C). Inhibition of the expression of hsa‐miR‐6817‐5p could partially reverse the suppressive effect of si‐circRFWD2 (Figure 5D) and si‐circINO80 (Figure 5E), respectively, on the expression of RUNX2.

## 4 DISCUSSION

CircRNAs are highly conserved among species due to the closed loop structures,^35^ and they could be divided into five patterns, including exonic circRNAs, intronic circRNAs, intergenic circRNAs, sense overlapping circRNAs and antisense circRNAs, according to the different formation mechanisms.^19^

It is reported that circRNAs could interact with RNA‐binding protein and affect the protein function.^18^ A few circRNAs could be directly translated into proteins.^36^ Exonic‐intronic circRNAs were associated with U1 small nuclear ribonucleoproteins, while intronic circRNAs were related to the polymerase II machinery, and both of them could regulate the host gene expression.^37^ In addition, multiple circRNAs were known to harbour the binding sites of miRNAs, making them competently bind to miRNAs and buffer the inhibition of miRNAs on target genes, which played a critical regulatory role in the osteogenesis of mesenchymal stem cells.^38^ Hsa_circRNA_33287 regulated the expression of Runx3 via targeting miR‐214‐3p to enhance the osteogenic differentiation of maxillary sinus membrane stem cells.^39^ Hsa_circ_0002052 targeted miR‐1205 to regulate the expression of APC2, which inhibited Wnt/beta‐catenin signalling pathway.^40^, ^41^ Mm9_circ_009056 could act as a sponge of miR‐22‐3p, regulating the expression of BMP7 to promote bone formation.^42^ CircRNA CDR1as could down‐regulate miR‐7 to activate p38 MAPK pathway, which enhanced the osteogenesis of periodontal ligament stem cells.^43^ However, the roles of circRNAs in recombinant NELL‐1‐induced osteogenesis of hASCs remains unclear.

In this study, we performed GO analysis to annotate biological functions of parental genes of differentially expressed circRNAs. The biological processes, in which most circRNAs were enriched, were the regulation of cell cycle process, the organelle organization and the mitotic nuclear division. The most enriched cellular components terms were the intracellular part, the nucleoplasm and the intracellular. For molecular function aspect, the most enriched terms were related to the protein binding, the guanyl‐nucleotide exchange factor activity and the small GTPase binding. The KEGG pathway analysis suggested most of the differentially expressed circRNAs were enriched in the Glutamatergic synapse, THs signalling pathway and p53 signalling pathway. p53 signalling pathway was reported to regulate bone formation negatively in vivo and in vitro, under the control of BMPs and IGFs pathways.^44^ It has shown that p53 could suppress the expression of osterix (Osx) to influence osteoblastic differentiation.^45^ Meanwhile, p53 could affect the cell fate of mouse bone marrow‐derived mesenchymal stem cells and activate miRNA‐34 family to repress the expression of Runx2.^46^ It was known that THs played roles in the endochondral ossification by affecting chondrocytes and osteoblasts directly, and regulated the initiation and progression of the secondary ossification centre through promoting the chondrocytes to differentiate into bone matrix‐producing osteoblasts via activating Indian hedgehog and Osx expression.^47^ NR2F1, one of the steroid/THs nuclear receptor superfamily members, could impair the osteoblast differentiation, which could be rescued by BMP‐2.^48^ Therefore, the circRNAs identified might influence the recombinant NELL‐1‐induced osteogenesis of hASCs via p53 signalling pathway and THs signalling pathway, cross‐talking with BMPs signalling pathway.

Additionally, circRFWD2 and circINO80 were identified to be up‐regulated during recombinant NELL‐1‐induced osteogenesis by RNA‐seq, which was validated by qRT‐PCR. When inhibiting circRFWD2 and circINO80, respectively, we found the osteogenic markers, RUNX2 and OPN, were down‐regulated obviously. The ALP activity and calcium nodules staining were further confirmed the results above. During recombinant NELL‐1‐induced osteogenesis, the expression of RUNX2 and OPN were also decreased significantly in both of circRFWD2 and circINO80 knockdown groups than group NC. Therefore, it is suggested that these two circRNAs might influence recombinant NELL‐1‐induced osteogenesis positively.

Our study showed that hsa‐miR‐6817‐5p was down‐regulated during the recombinant NELL‐1‐induced osteogenesis, and it could inhibit the recombinant NELL‐1‐induced osteogenic differentiation. It is suggested that hsa‐miR‐6817‐5p was closely associated with hsa_circ_0061012, which was up‐regulated in psoriasis significantly. Psoriasis was related well to the regulation of NF‐kappaB,^49^ whose activity could be weakened by NELL‐1.^50^

Moreover, we found that the binding sites of hsa‐miR‐6817‐5p could match the sequences of circRFWD2 and circINO80, respectively, and the expression levels of these two circRNAs were negatively correlated with hsa‐miR‐6817‐5p. Inhibition of hsa‐miR‐6817‐5p expression could partially reverse the suppressive effect of si‐circRFWD2 and si‐circINO80 on the osteogenesis. It is suggested that circRFWD2 and circINO80 could regulate hsa‐miR‐6817‐5p and promote recombinant NELL‐1‐induced osteogenesis of hASCs.

Although we tried the best to design our experiments, there are some limitations in this study. It was difficult to obtain the clinical samples of unilateral coronal synostosis, where NELL‐1 was detected to be over‐expressed, and thus, we did not verify the expression profiles of CircRFWD2 and circINO80 axis in the clinical specimen data. Furthermore, some interesting and valuable research issues, for example, whether and how NELL‐1 could regulate miR‐6817‐5p, are need to be studied. Therefore, we would focus on miming and enriching the mechanism of circRFWD2 and circINO80 axis in recombinant NELL‐1‐induced osteogenesis, and this study opened the window.

## 5 CONCLUSION

circRFWD2 and circINO80 were identified to up‐regulated during the recombinant NELL‐1‐induced osteogenesis. Mechanistically, circRFWD2 and circINO80 could regulate hsa‐miR‐6817‐5p and influence recombinant NELL‐1‐induced osteogenic differentiation of hASCs. Therefore, circRFWD2 and circINO80 promise to be the potential molecular targets for the regulation of osteogenesis and bone regeneration.

## CONFLICT OF INTEREST

The authors declare that there are no conflicts of interest.

## AUTHOR CONTRIBUTIONS

X. H., J. L. and Z. Z. designed the research. X. H., X. C., B. Z. and G. Z. conducted the research. Y. L. and J. L. contributed to figures and linguistic revision. X. H. and X. C. wrote the paper. All authors reviewed the results and approved the final version of the manuscript.

## Data Availability

The data that support the findings of this study are available from the corresponding author upon reasonable request.

## References

[1] Zhang X , Zara J , Siu RK , et al. The role of NELL‐1, a growth factor associated with craniosynostosis, in promoting bone regeneration. J Dent Res. 2010;89(9):865‐878. 20647499 10.1177/0022034510376401 PMC2959101

[2] Canady JW , Zeitler DP , Thompson SA , Nicholas CD . Suitability of the iliac crest as a site for harvest of autogenous bone grafts. Cleft Palate Craniofac J. 1993;30(6):579‐581. 8280737 10.1597/1545-1569_1993_030_0579_sotica_2.3.co_2

[3] Frodel JL Jr , Marentette LJ , Quatela VC , Weinstein GS . Calvarial bone graft harvest. Techniques, considerations, and morbidity. Arch Otolaryngol Head Neck Surg. 1993;119(1):17‐23. 8417739 10.1001/archotol.1993.01880130019002

[4] Agarwal R , García AJ . Biomaterial strategies for engineering implants for enhanced osseointegration and bone repair. Adv Drug Deliv Rev. 2015;94:53‐62. 25861724 10.1016/j.addr.2015.03.013 PMC4598264

[5] Gruskin E , Doll BA , Futrell FW , et al. Demineralized bone matrix in bone repair: history and use. Adv Drug Deliv Rev. 2012;64(12):1063‐1077. 22728914 10.1016/j.addr.2012.06.008 PMC7103314

[6] O'Keefe RJ , Mao J . Bone tissue engineering and regeneration: from discovery to the clinic–an overview. Tissue Eng Part B Rev. 2011;17(6):389‐392. 21902614 10.1089/ten.teb.2011.0475 PMC3223012

[7] Chen W , Liu J , Manuchehrabadi N , et al. Umbilical cord and bone marrow mesenchymal stem cell seeding on macroporous calcium phosphate for bone regeneration in rat cranial defects. Biomaterials. 2013;34(38):9917‐9925. 24054499 10.1016/j.biomaterials.2013.09.002 PMC4023544

[8] De GL , Sartori MF , Arrigoni E , et al. Human adipose‐derived stem cells as future tools in tissue regeneration: osteogenic differentiation and cell‐scaffold interaction. Int J Artif Organs. 2008;31(6):467. 18609499 10.1177/039139880803100602

[9] Liao HT , Chen CT . Osteogenic potential: comparison between bone marrow and adipose‐derived mesenchymal stem cells. World J Stem Cells. 2014;6(3):288‐295. 25126378 10.4252/wjsc.v6.i3.288 PMC4131270

[10] Ting K , Vastardis H , Mulliken JB , et al. Human NELL‐1 expressed in unilateral coronal synostosis. J Bone Miner Res. 1999;14(1):80‐89. 9893069 10.1359/jbmr.1999.14.1.80

[11] Desai J , Shannon ME , Johnson MD , et al. Nell1‐deficient mice have reduced expression of extracellular matrix proteins causing cranial and vertebral defects. Hum Mol Genet. 2006;15(8):1329‐1341. 16537572 10.1093/hmg/ddl053

[12] Liu J , Chen W , Zhao Z , Xu H . Effect of NELL1 gene overexpression in iPSC‐MSCs seeded on calcium phosphate cement. Acta Biomater. 2014;10(12):5128‐5138. 25220281 10.1016/j.actbio.2014.08.016 PMC4559224

[13] Liu Y , Chen C , He H , et al. Lentiviral‐mediated gene transfer into human adipose‐derived stem cells: role of NELL1 versus BMP2 in osteogenesis and adipogenesis *in vitro* . Acta Biochim Biophys Sin. 2012;44(10):856‐865. 23017834 10.1093/abbs/gms070

[14] Zhu S , Zhang B , Man C , et al. NEL‐like molecule‐1‐modified bone marrow mesenchymal stem cells/poly lactic‐co‐glycolic acid composite improves repair of large osteochondral defects in mandibular condyle. Osteoarthritis Cartilage. 2011;19(6):743‐750. 21362490 10.1016/j.joca.2011.02.015

[15] Zhang X , Kuroda S , Carpenter D , et al. Craniosynostosis in transgenic mice overexpressing Nell‐1. J Clin Invest. 2002;110(6):861‐870. 12235118 10.1172/JCI15375 PMC151127

[16] Cowan CM , Jiang X , Hsu T , et al. Synergistic effects of Nell‐1 and BMP‐2 on the osteogenic differentiation of myoblasts. J Bone Miner Res. 2007;22(6):918‐930. 17352654 10.1359/jbmr.070312 PMC2866074

[17] Zhang X , Ting K , Bessette CM , et al. Nell‐1, a key functional mediator of Runx2, partially rescues calvarial defects in Runx2(+/−) mice. J Bone Miner Res. 2011;26(4):777‐791. 20939017 10.1002/jbmr.267 PMC3179324

[18] Qu S , Yang X , Li X , et al. Circular RNA: a new star of noncoding RNAs. Cancer Lett. 2015;365(2):141‐148. 26052092 10.1016/j.canlet.2015.06.003

[19] Wilusz JE , Sharp PA . Molecular biology. A circuitous route to noncoding RNA. Science. 2013;340(6131):440‐441. 23620042 10.1126/science.1238522 PMC4063205

[20] Chen LL , Yang L . Regulation of circRNA biogenesis. RNA Biol. 2015;12(4):381‐388. 25746834 10.1080/15476286.2015.1020271 PMC4615371

[21] Xia S , Feng J , Chen K , et al. CSCD: a database for cancer‐specific circular RNAs. Nucleic Acids Res. 2018;46(D1):D925‐D929. 29036403 10.1093/nar/gkx863 PMC5753219

[22] Lasda E , Parker R . Circular RNAs: diversity of form and function. RNA. 2014;20(12):1829‐1842. 25404635 10.1261/rna.047126.114 PMC4238349

[23] Greco S , Cardinali B , Falcone G , Martelli F . Circular RNAs in muscle function and disease. Int J Mol Sci. 2018;19(11):3454. 10.3390/ijms19113454 PMC6274904 30400273

[24] Carrara M , Fuschi P , Ivan C , Martelli F . Circular RNAs: methodological challenges and perspectives in cardiovascular diseases. J Cell Mol Med. 2018;22(11):5176‐5187. 30277664 10.1111/jcmm.13789 PMC6201346

[25] E S , Costa MC , Kurc S , et al. The circulating non‐coding RNA landscape for biomarker research: lessons and prospects from cardiovascular diseases. Acta Pharmacol Sin. 2018;39(7):1085‐1099. 29877319 10.1038/aps.2018.35 PMC6289369

[26] Cai H , Li Y , Niringiyumukiza JD , et al. Circular RNA involvement in aging: an emerging player with great potential. Mech Ageing Dev. 2018;178:16‐24. 30513309 10.1016/j.mad.2018.11.002

[27] Li LJ , Zhu ZW , Zhao W , et al. Circular RNA expression profile and potential function of hsa_circ_0045272 in systemic lupus erythematosus. Immunology. 2018;155(1):137‐149. 29700819 10.1111/imm.12940 PMC6099170

[28] Chen D , Ma W , Ke Z , Xie F . CircRNA hsa_circ_100395 regulates miR‐1228/TCF21 pathway to inhibit lung cancer progression. Cell Cycle. 2018;17(16):2080‐2090. 30176158 10.1080/15384101.2018.1515553 PMC6224268

[29] He JH , Han ZP , Zhou JB , et al. MiR‐145 affected the circular RNA expression in prostate cancer LNCaP cells. J Cell Biochem. 2018;119(11):9168‐9177. 30136305 10.1002/jcb.27181 PMC6704360

[30] Guan Z , Tan J , Gao W , et al. Circular RNA hsa_circ_0016788 regulates hepatocellular carcinoma tumorigenesis through miR‐486/CDK4 pathway. J Cell Physiol. 2018;234(1):500‐508. 29923236 10.1002/jcp.26612

[31] Zhang Y , Liang W , Zhang P , et al. Circular RNAs: emerging cancer biomarkers and targets. J Exp Clin Cancer Res. 2017;36(1):152. 29096676 10.1186/s13046-017-0624-z PMC5667461

[32] Floris G , Zhang L , Follesa P , Sun T . Regulatory role of circular RNAs and neurological disorders. Mol Neurobiol. 2017;54(7):5156‐5165. 27558238 10.1007/s12035-016-0055-4 PMC5955391

[33] James AW , Pan A , Chiang M , et al. A new function of Nell‐1 protein in repressing adipogenic differentiation. Biochem Biophys Res Commun. 2011;411(1):126‐131. 21723263 10.1016/j.bbrc.2011.06.111 PMC3166249

[34] Robinson MD , McCarthy DJ , Smyth GK . edgeR: a bioconductor package for differential expression analysis of digital gene expression data. Bioinformatics. 2010;26(1):139‐140. 19910308 10.1093/bioinformatics/btp616 PMC2796818

[35] Jeck WR , Sorrentino JA , Wang K , et al. Circular RNAs are abundant, conserved, and associated with ALU repeats. RNA. 2013;19(2):141‐157. 23249747 10.1261/rna.035667.112 PMC3543092

[36] Li Z , Huang C , Bao C , et al. Exon‐intron circular RNAs regulate transcription in the nucleus. Nat Struct Mol Biol. 2015;22(3):256‐264. 25664725 10.1038/nsmb.2959

[37] Hansen TB , Jensen TI , Clausen BH , et al. Natural RNA circles function as efficient microRNA sponges. Nature. 2013;495(7441):384‐388. 23446346 10.1038/nature11993

[38] Chen CY , Sarnow P . Initiation of protein synthesis by the eukaryotic translational apparatus on circular RNAs. Science. 1995;268(5209):415‐417. 7536344 10.1126/science.7536344

[39] Peng W , Zhu S , Chen J , et al. Hsa_circRNA_33287 promotes the osteogenic differentiation of maxillary sinus membrane stem cells via miR‐214‐3p/Runx3. Biomed Pharmacother. 2019;109:1709‐1717. 30551425 10.1016/j.biopha.2018.10.159

[40] Wu Z , Shi W , Jiang C . Overexpressing circular RNA hsa_circ_0002052 impairs osteosarcoma progression via inhibiting Wnt/beta‐catenin pathway by regulating miR‐1205/APC2 axis. Biochem Biophys Res Commun. 2018;502(4):465‐471. 29852168 10.1016/j.bbrc.2018.05.184

[41] Gong YY , Peng MY , Yin DQ , Yang YF . Long non‐coding RNA H19 promotes the osteogenic differentiation of rat ectomesenchymal stem cells via Wnt/beta‐catenin signaling pathway. Eur Rev Med Pharmacol Sci. 2018;22(24):8805‐8813. 30575922 10.26355/eurrev_201812_16648

[42] Wu C , Zheng Z , Ren W , et al. Mm9_circ_009056 enhances osteogenesis by targeting BMP7 via CGRP‐mediated miR‐22‐3p. Biochem Biophys Res Commun. 2018;501(1):199‐205. 29709471 10.1016/j.bbrc.2018.04.215

[43] Li X , Zheng Y , Zheng Y , et al. Circular RNA CDR1as regulates osteoblastic differentiation of periodontal ligament stem cells via the miR‐7/GDF5/SMAD and p38 MAPK signaling pathway. Stem Cell Res Ther. 2018;9(1):232. 30170617 10.1186/s13287-018-0976-0 PMC6119336

[44] Huijuan L , Baojie L . p53 control of bone remodeling. J Cell Biochem. 2010;111(3):529‐534. 20589754 10.1002/jcb.22749

[45] Wang X , Kua HY , Hu Y , et al. p53 functions as a negative regulator of osteoblastogenesis, osteoblast‐dependent osteoclastogenesis, and bone remodeling. J Cell Biol. 2006;172(1):115‐125. 16380437 10.1083/jcb.200507106 PMC2063539

[46] He Y , de Castro LF , Shin MH , et al. p53 loss increases the osteogenic differentiation of bone marrow stromal cells. Stem Cells. 2015;33(4):1304‐1319. 25524638 10.1002/stem.1925 PMC4376591

[47] Weirong X , Shaohong C , Jon W , Subburaman M . Epiphyseal chondrocyte secondary ossification centers require thyroid hormone activation of Indian hedgehog and osterix signaling. J Bone Miner Res. 2015;29(10):2262‐2275. 10.1002/jbmr.2256 PMC4487616 24753031

[48] Manikandan M , Abuelreich S , Elsafadi M , et al. NR2F1 mediated down‐regulation of osteoblast differentiation was rescued by bone morphogenetic protein‐2 (BMP‐2) in human MSC. Differentiation. 2018;104:36‐41. 30445268 10.1016/j.diff.2018.10.003

[49] Qiao M , Ding J , Yan J , et al. Circular RNA expression profile and analysis of their potential function in psoriasis. Cell Physiol Biochem. 2018;50(1):15‐27. 30278433 10.1159/000493952

[50] Shen J , James AW , Zara JN , et al. BMP2‐induced inflammation can be suppressed by the osteoinductive growth factor NELL‐1. Tissue Eng Part A. 2013;19(21–22):2390‐2401. 23758588 10.1089/ten.tea.2012.0519 PMC3807546

