# Supplementary figures and images for: The roles of circRFWD2 and circINO80 during NELL‐1‐induced osteogenesis
Source: J Cell Mol Med. 2019 Oct 21;23(12):8432–41. doi: 10.1111/jcmm.14726 (PMC6850935; doi:10.1111/jcmm.14726)

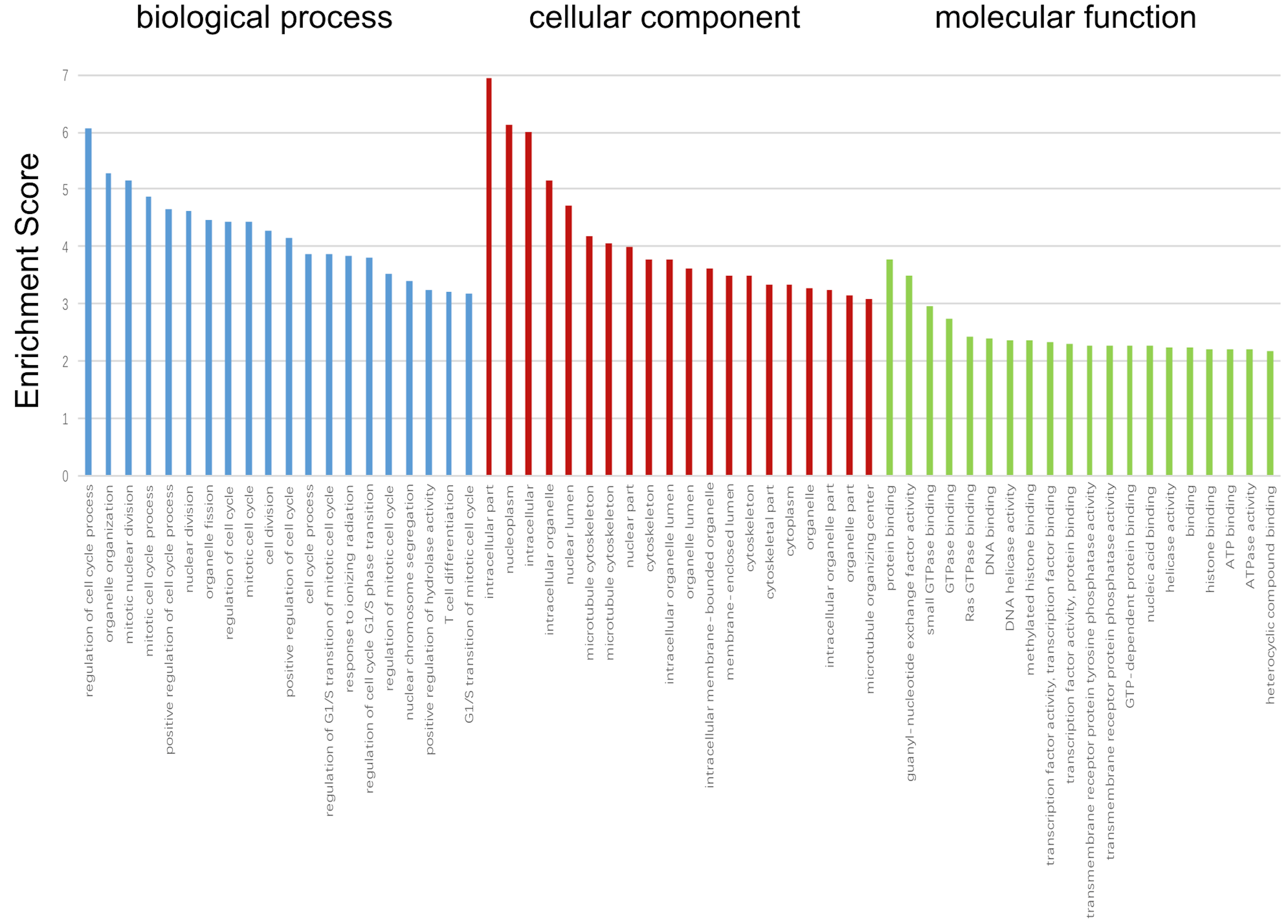

Supplement: Supplementary file 1 [file JCMM-23-8432-s001.tiff]

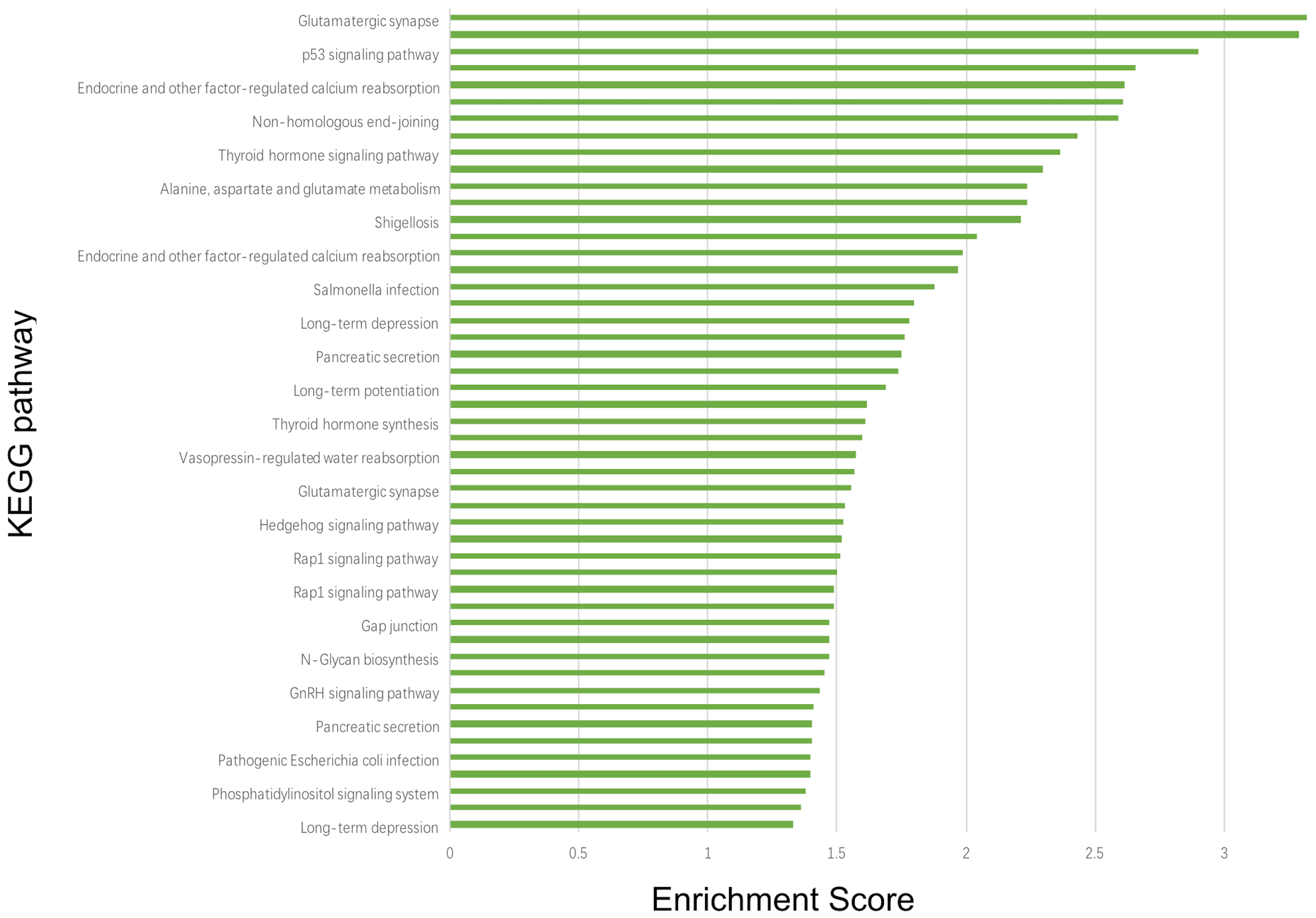

Supplement: Supplementary file 2 [file JCMM-23-8432-s002.tiff]
