# Supplementary material for: The roles of circRFWD2 and circINO80 during NELL‐1‐induced osteogenesis
Source: J Cell Mol Med. 2019 Oct 21;23(12):8432–41. doi: 10.1111/jcmm.14726 (PMC6850935; doi:10.1111/jcmm.14726)
Supplement: Supplementary file 3 [file JCMM-23-8432-s003.docx]

**TABLE S1** The sequences of the RNA oligoribonucleotide

|  | Sequence |
| --- | --- |
| hsa-miR-6817-5p mimic | UCUGCCAUAGGAAGCUUGGAGUGG |
| hsa-miR-6817-5p inhibitor | CCACUCCAAGCUUCCUAUGGCAGA |
| miR-NC | Forward: 5’-UUGUACUACACAAAAGUACUG -3’ |
| si-circINO80-1 | Forward: 5’-CUGCUAUUGAUGAGAUGUUTT-3’  Reverse: 5’- AACAUCUCAUCAAUAGCAGTT-3’ |
| si-circINO80-2 | Forward: 5’-GAUGAGAUGUUCGUUGGAATT-3’  Reverse: 5’- UUCCAACGAACAUCUCAUCTT-3’ |
| si-circINO80-3 | Forward: 5’-UAUUGAUGAGAUGUUCGUUTT-3’  Reverse: 5’- AACGAACAUCUCAUCAAUATT-3’ |
| si-circRFWD2-1 | Forward: 5’-CAGUAUAGCCUCUAGUAGUTT-3’  Reverse: 5’- ACUACUAGAGGCUAUACUGTT-3’ |
| si-circRFWD2-2 | Forward: 5’-CUCUAGUAGUAUUAUGAUCTT-3’  Reverse: 5’- GAUCAUAAUACUACUAGAGTT-3’ |
| si-circRFWD2-3 | Forward: 5’-UAGCCUCUAGUAGUAUUAUTT-3’  Reverse: 5’- AUAAUACUACUAGAGGCUATT-3’ |
| si-NC | Forward: 5'- UUCUCCGAACGUGUCACGUTT -3'  Reverse: 5'- ACGUGACACGUUCGGAGAATT -3' |

**TABLE S2** Primer sequences list of qRT-PCR

|  | Primer sequences (5’-3’) |
| --- | --- |
| circRFWD2 | F: TGCAAGCCAGTTGGATGA  R: AAACCTGGAGGTTGGCTG |
| circINO80 | F: AGCCATGCCGAAAACAAA  R: TAGCAAAAGCCGATTCCG |
| circDCBLD2 | F: TGGGAGAGAGAGTTCGCA  R: GGGTTCCACTCTCAGGGC |
| circHAGH | F: CTGCACCTTCAGCGGATT  R: GCACCGTCTTCTCCCTCA |
| RUNX2 | F: CTACTATGGCACTTCGTCAGGAT  R: ATCAGCGTCAACACCATCATT |
| COL I | F: CCAAGACGAAGACATCCCACCA  R: CCGTTGTCGCAGACGCAGAT |
| NELL-1 | F: GGTGTCTGGAATGCACAATG  R: AAGTGGATGGCTTCTGCTGT |
| OPN | F: GGCTAAACCCTGACCCATCTC  R: GTCAATGGAGTCCTGGCTGTC |
| β-ACTIN | F: CACCCAGCACAATGAAGATCAAGAT  R: CCAGTTTTTAAATCCTGAGTCAAGC |
