# Supplementary material for: The roles of circRFWD2 and circINO80 during NELL‐1‐induced osteogenesis
Source: J Cell Mol Med. 2019 Oct 21;23(12):8432–41. doi: 10.1111/jcmm.14726 (PMC6850935; doi:10.1111/jcmm.14726)
Supplement: Supplementary file 4 [file JCMM-23-8432-s004.doc]

**Figure S1** GO analysis.

**FIGURE S2** KEGG pathway analysis.
